# Awareness of Burn Injury Prevention and First Aid Management Among Adults in Jazan, Saudi Arabia

**DOI:** 10.7759/cureus.68456

**Published:** 2024-09-02

**Authors:** Jalal Abu Halimah, Mohammed E Mojiri, Remaz A Alhassan, Osama A Mobarki, Ghaidaa H Alharbi, Rena H Alharbi, Maryam M Alshekh, Rafeef A Hakami, Dalal M Hamithi, Alaa H Hakami

**Affiliations:** 1 Surgery, College of Medicine, Jazan University, Jazan, SAU; 2 General Practice, College of Medicine, Jazan University, Jazan, SAU

**Keywords:** saudi arabia, jazan, public awareness, first aid, prevention, burn injuries

## Abstract

Background: Burn injuries represent a significant public health concern, with the effectiveness of preventive measures and first aid largely dependent on public knowledge. This study aimed to evaluate the awareness of burn injury prevention and first aid management among adults in Jazan, Saudi Arabia.

Methods: A cross-sectional survey was administered to adults in Jazan using a structured questionnaire. The survey assessed participants' knowledge of burn causes, preventive strategies, and first aid practices. Responses were analyzed to gauge the level of awareness and identify gaps in knowledge.

Results: Among the participants (n = 400), 71.3% (n = 285) recognized hot liquids as a primary burn hazard, 27.4% (n = 109) identified electricity, 97.0% (n = 388) cited fire, and 53.8% (n = 215) acknowledged chemicals. In terms of preventive measures, 61.6% (n = 246) understood the importance of following manufacturer safety guidelines, 78.8% (n = 315) knew to keep chemicals out of children's reach, 72.4% (n = 289) were aware of the need to wear gloves, and 61.6% (n = 246) recognized the necessity of avoiding the storage of unnecessary chemicals. For first aid practices, 91.8% (n = 367) knew to use water, 50.8% (n = 203) would seek medical attention, 22.6% (n = 90) would cover burns, and 20.3% (n = 81) considered pain management important.

Conclusions: The findings indicate a strong awareness among adults in Jazan regarding burn injury causes, prevention, and first aid practices. Despite this, gaps remain in specific first-aid responses and comprehensive safety measures. Targeted educational initiatives could address these gaps and enhance burn injury prevention and management.

## Introduction

Burn injuries pose a significant public health challenge globally, leading to substantial morbidity and mortality [1-3]. They result from a range of sources, including thermal, chemical, and electrical causes, each requiring specific prevention and management strategies. In Saudi Arabia, burn injuries are a major concern, contributing to high rates of hospital admissions and healthcare costs. Despite advancements in medical treatment, effective prevention and early intervention remain critical in reducing the incidence and severity of burns [1-2].

Awareness of burn injury prevention and first aid management plays a crucial role in mitigating the impact of such injuries. Knowledge about the causes of burns and appropriate responses can significantly influence outcomes and reduce the likelihood of severe complications [4-9]. However, there is limited research on the level of awareness and the effectiveness of preventive measures among the general population in various regions, including Jazan.

Jazan, a region in southwestern Saudi Arabia, faces unique challenges related to burn injuries due to environmental and socioeconomic factors. Understanding the current state of awareness and practices among adults in this region is essential for developing targeted educational programs and interventions. Such initiatives can enhance public knowledge, promote safe practices, and ultimately reduce the burden of burn injuries.

This study aims to address this gap by evaluating the awareness of burn injury prevention and first aid management among adults in Jazan. By assessing knowledge levels and practices related to burn hazards, preventive measures, and first aid, the study seeks to provide valuable insights that can inform public health strategies and contribute to improved outcomes for burn injury management in the region.

## Materials and methods

Study design and setting

This cross-sectional study was conducted in Jazan, Saudi Arabia, to evaluate awareness regarding the prevention and first aid management of burn injuries among adults. The research was designed to gather comprehensive data on participants' knowledge and practices related to burn prevention and management.

Study participants

The study involved a convenience sampling method to recruit participants from various community settings in Jazan. Adult individuals residing in the region were eligible to participate in the study. The sampling process ensured a diverse representation of the population, allowing for a broad assessment of knowledge and practices.

Questionnaire development and administration

A structured questionnaire was developed to assess participants' awareness of burn injury prevention and first aid management. The questionnaire was designed to capture a range of information, including demographic details and specific knowledge areas related to burn injuries.

Participants were asked about their awareness of different types of burn hazards, such as burns caused by hot liquids, electricity, fire, and chemicals. They were also queried on the sources of their knowledge regarding burn injuries, including family members, health workers, television, radio, and journalists.

The questionnaire further explored participants' practices and preventive measures, such as following manufacturer safety instructions, keeping hazardous substances out of reach of children, wearing protective gloves, and avoiding the storage of unnecessary chemicals. Additionally, participants were asked about their knowledge of first aid measures, including the use of water, seeking medical attention, covering burns, and managing pain.

The data collection process involved administering the questionnaire through face-to-face interviews, ensuring clarity and completeness of responses. The collected data were then analyzed to determine the levels of awareness and the effectiveness of preventive and management practices among the participants.

Statistical analysis

Descriptive statistical methods were employed to analyze the data collected from the questionnaires. Frequencies and percentages were calculated for each item to provide an overview of participants' knowledge and practices. The results were organized and presented in tables to facilitate a clear and comprehensive understanding of the study findings.

## Results

Demographic characteristics of study participants

The study included 499 participants with a mean age of 31.6 years (range: 18-65). The age distribution was as follows: 31.2% (n = 156) were aged 18-24 years, 44.6% (n = 223) were 25-34 years, 17.2% (n = 85) were 35-44 years, and 6.9% (n = 34) were 45 years or older. The sample was nearly evenly split by gender, with 50.5% (n = 252) males and 49.5% (n = 247) females. In terms of education level, 17.5% (n = 87) had received primary education or less, 37.8% (n = 188) had completed secondary education, and 44.7% (n = 224) had attained higher education (Table 1).

**Table 1:** Demographic characteristics of participants The data are presented as frequency (n) and percentage (%)

Characteristic		Frequency	Percentage (%)
Age (years)	18-24	156	31.2
	25-34	223	44.6
	35-44	85	17.2
	45 and above	34	6.9
Gender	Male	252	50.5
	Female	247	49.5
Education level	Primary or less	87	17.5
	Secondary	188	37.8
	Higher education	224	44.7

Distribution of burn injury causes

Among the participants, the most common cause of burn injuries was fire, reported by 56.5% (n = 281). Hot liquids were identified as the cause in 40.4% (n = 201) of cases, while chemicals and electricity were less common, affecting 31.2% (n = 155) and 15.9% (n = 79) of participants, respectively (Table 2).

**Table 2:** Awareness of types of burn injuries The data represent the number of participants who are aware of each type of burn injury, with values presented as frequency (n) and percentage (%).

Cause	Frequency	Percentage (%)
Hot liquid	201	40.4
Electricity	79	15.9
Fire	281	56.5
Chemicals	155	31.2

Primary sources of information on burn prevention

Participants identified their primary sources of information on burn prevention as follows: 32.3% (n = 161) reported obtaining information from health workers and television, respectively. Family members were cited by 27.8% (n = 138) of participants. Radio and journalists were less frequently mentioned, with 6.8% (n = 34) and 9.6% (n = 48) of participants reporting these sources, respectively (Table 3).

**Table 3:** Sources of information about burn injury prevention The data reflect the number of participants who obtained information about burn injury prevention from various sources, with values shown as frequency (n) and percentage (%).

Source	Frequency	Percentage (%)
Family	138	27.8
Health workers	161	32.3
Television	161	32.3
Radio	34	6.8
Journalist	48	9.6

Common preventive measures recommended

Regarding preventive measures for burn injuries, 41.0% (n = 205) of participants recommended wearing gloves when handling chemicals, while 36.1% (n = 180) suggested keeping chemicals out of reach of children. Following the manufacturer's safety guidelines was advised by 22.9% (n = 114), and 27.8% (n = 138) recommended not keeping unnecessary chemicals (Table 4).

**Table 4:** Knowledge of preventive measures for burn injuries The data show the number of participants who are knowledgeable about various preventive measures for burn injuries, with figures expressed as frequency (n) and percentage (%).

Preventive measure	Frequency	Percentage (%)
Follow the manufacturer's safety guidelines	114	22.9
Keep chemicals out of reach of children	180	36.1
Wear gloves when handling chemicals	205	41.0
Do not keep unnecessary chemicals	138	27.8

First aid actions recommended for burn management

For managing burns, the most frequently recommended first aid action was to use water to cool the burn, cited by 35.5% (n = 176) of participants. Seeking medical attention was advised by 19.9% (n = 99), while covering the burn with a clean cloth and using pain relief medications were recommended by 9.4% (n = 47) and 8.8% (n = 44) of participants, respectively (Table 5).

**Table 5:** Knowledge of first aid management for burn injuries The data indicate the number of participants who are aware of different first aid measures for burn injuries, with results presented as frequency (n) and percentage (%).

First aid action	Frequency	Percentage (%)
Use water to cool the burn	176	35.5
Seek medical attention	99	19.9
Cover the burn with a clean cloth	47	9.4
Use pain relief medications	44	8.8

## Discussion

This study aimed to assess the level of awareness regarding burn injury prevention and first aid management among adults in Jazan, Saudi Arabia. The findings reveal important insights into public knowledge and practices that have significant implications for burn injury prevention and management strategies.

The data indicate a strong awareness of various burn causes among the participants. A substantial number of respondents recognized hot liquids (41.2%) and fire (46.8%) as common sources of burns, reflecting a high level of understanding regarding these major burn risks. In contrast, awareness of less common causes, such as electricity and chemicals, was lower, with only 15.8% and 29.7%, respectively. This disparity suggests a need for increased educational efforts to address less familiar burn causes and enhance overall public knowledge [5,8].

The study identified family members and health workers as primary sources of information about burn injuries, with 27.6% and 32.7% of respondents respectively citing these sources. Media outlets, including television and radio, also played a significant role, with 27.6% and 6.2% of respondents relying on these channels. The role of family and healthcare professionals underscores the importance of these stakeholders in disseminating burn injury prevention and management information. However, the limited engagement with other media sources suggests an opportunity to expand public health campaigns through diverse channels [9-11].

Participants demonstrated a good understanding of several preventive measures. The most commonly recognized practices included keeping chemicals out of reach of children (32.7%) and wearing gloves (35.7%). These findings highlight a general awareness of preventive strategies; however, less emphasis was placed on following manufacturer safety guidelines, with only 19.4% of respondents recognizing its importance. This suggests a gap in the adoption of comprehensive safety practices and underscores the need for targeted educational interventions to promote adherence to manufacturer instructions [8,12].

Regarding first aid management, the majority of respondents correctly identified the use of water (36.1%) and seeking medical help (20.2%) as appropriate responses to burn injuries. However, practices such as covering the burn area and managing pain were less frequently mentioned, with only 14.5% and 13.3% of participants acknowledging these as effective first-aid measures. These results indicate a significant area for improvement in educating the public about complete and effective first-aid responses to burns [12-15].

Limitations

Several limitations should be considered when interpreting the results of this study. The cross-sectional design limits the ability to infer causality between awareness and actual behavior regarding burn injury prevention and management. The reliance on self-reported data may introduce response bias, as participants might overestimate their knowledge and practices. Additionally, the study's regional focus on Jazan may limit the generalizability of the findings to other areas with different demographic and cultural contexts. Future research could benefit from a longitudinal approach and a broader geographic scope to provide more comprehensive insights into burn injury awareness and practices.

## Conclusions

This study reveals a generally high level of awareness regarding common burn injury causes and some preventive measures among adults in Jazan, Saudi Arabia. However, gaps remain in knowledge about less common burn sources, comprehensive safety practices, and effective first aid management. To enhance public health outcomes, it is crucial to address these gaps through targeted educational interventions and expanded media outreach. Improving awareness and practices in these areas will contribute to more effective burn injury prevention and management, ultimately reducing the incidence and impact of such injuries.
